# Molecular Characterization and Antimicrobial Susceptibility of *C. jejuni* Isolates from Italian Wild Bird Populations

**DOI:** 10.3390/pathogens9040304

**Published:** 2020-04-20

**Authors:** Francesca Marotta, Anna Janowicz, Lisa Di Marcantonio, Claudia Ercole, Guido Di Donato, Giuliano Garofolo, Elisabetta Di Giannatale

**Affiliations:** 1 Istituto Zooprofilattico Sperimentale dell’Abruzzo e del Molise “G. Caporale”, National Reference Laboratory for Campylobacter, 64100 Teramo, Italy; a.janowicz@izs.it (A.J.); l.dimarcantonio@izs.it (L.D.M.); guido.didonato@live.it (G.D.D.); g.garofolo@izs.it (G.G.); e.digiannatale@izs.it (E.D.G.); 2 Department of Life, Health and Environmental Sciences, University of L’Aquila, 67100 L’Aquila, Italy; claudia.ercole@univaq.it

**Keywords:** *Campylobacter jejuni*, antimicrobial resistance, multidrug resistance, MLST, cgMLST, AMR genes

## Abstract

Poultry is considered a major reservoir of human campylobacteriosis. It also been reported that not only poultry, but also wild birds, are capable of carrying *C. jejuni*, thus demonstrating to be a risk of spreading the bacteria in the environment. To gain insight into the population structure and investigate the antimicrobial resistance genotypes and phenotypes, we analyzed a collection of 135 *C. jejuni* from 15 species of wild birds in Italy. MLST revealed the presence of 41 sequence types (STs) and 13 clonal complexes (CCs). ST-179 complex and the generalist ST-45 complex were the most prevalent. Core genome MLST revealed that *C. jejuni* from ST-45 complex clustered according to the bird species, unlike the ST-179 complex which featured 3 different species in the same cluster. Overall we found a moderate prevalence of resistance to tetracycline (12.5%), ciprofloxacin and nalidixic acid (10%). The novel ST isolated from one pigeon showed resistance to all the antibiotics tested. The ST-179 complex (33.3%) was identified with significantly higher nalidixic acid resistance relative to other tested STs. Nine AMR genes (*tet*(O), *cme*A, *cme*B, *cme*C, *cme*R, aad, *bla*OXA-61, *bla*OXA-184 and *erm(B)*) and 23S rRNA and *gyr*A-associated point mutations were also described, indicating a concordance level between genotypic and phenotypic resistance of 23.3%, 23.4% and of 37.5% for streptomycin, tetracycline and quinolones/fluoroquinolones, respectively. We recommend that particular attention should be given to wild birds as key sentinel animals for the ecosystem contamination surveillance.

## 1. Introduction

Campylobacter disease continues to be the leading cause of bacterial gastroenteritis worldwide with a significant economic impact [1,2]. In human patients, apart from acute gastroenteritis, campylobacteriosis may lead to more severe, occasionally long-term sequelae, such as Guillain-Barre’ syndrome, reactive arthritis, and irritable bowel syndrome [3,4]. It is well known that most livestock species, carry *C.jejuni* asymptomatically [5], making control at the farm level difficult. However, some clones may cause gastroenteritis and abortion in bovine or sheep [6]. It is estimated that 50–80% of strains causing the disease in humans derived from the chicken reservoir, 20–30% from the cattle reservoir, and the remaining part originated from other reservoirs, including environment and wild animals [7]. Numerous findings suggest that wild birds, given their ability to fly long distances and their ubiquity, play an important role in the epidemiology and evolution of *Campylobacter* spp. [8,9]. In addition, some wild bird species have successfully adapted to anthropogenic environments and routinely come into close contact with livestock, domestic animals, and people [9]. They are, therefore, considered as reservoirs of farm animals and human infection [10,11]. In industrialized countries *C. jejuni* represents the major cause of human bacterial gastroenteritis [12] and seems to be the most prevalent species of *Campylobacter* isolated in wild birds [13,14,15,16]. It was observed that wild birds can carry different lineages of *C. jejuni* [17]; however, it is not yet completely clear if they play a role in the spread of *Campylobacter* strains to other hosts or can directly contribute to human disease acting as natural reservoirs of pathogenic genotypes [18]. Controversial data about *Campylobacter* strains isolated from wild birds potentially pathogenic for humans are known [18,19,20,21,22,23]. 

Transmission of *C. jejuni* from wild birds to humans can occur by direct contact, through contaminated food or water, or indirect contact. Many public places, are natural habitats for wild birds (mainly pigeons, starling or magpie), thus representing a risk of infection especially for people that spending their time at these public resting places [24]. Molecular studies, showed some genotypes strictly associated to a specific reservoir [25,26]. However, these host association studies have identified not only host-associated CCs but also multi-host associated CCs, with some genotypes overlapping wild birds, farm animals, poultry and human disease isolates [26,27]. Phylogenetically distinct lineages of *C. jejuni* in several wild bird species have been identified, sometimes with a host association particularly strong [17,25,28]. Furthermore, some authors reported that the association between *Campylobacter* genotypes and host wild bird species seems to overtake that between genotypes and geographic origin [17,29]. Multilocus sequence typing (MLST) is an effective molecular typing tool providing a more comprehensive knowledge of *C. jejuni* STs circulating. However, MLST is limited only to the characterization of the STs of the isolates [23,27,30]. In this context, more accurate methods that permit to compare genetically related isolated, such as whole or core-genome MLST (wgMLST, cgMLST), are increasingly being used [27,31]. Despite many studies conducted until now, the structure population of *C. jejuni* isolated from wild birds, the species-specific characteristics or the temporal or geographic stability are not yet fully understood. 

Antimicrobial resistance, a high-priority global health challenge, has been increasingly in wild birds, although they are not directly exposed to clinically relevant antimicrobials. Wild animals might play a vital role in the worldwide spread of clinically relevant pathogens or resistance genes. This underlies the complexity of bacterial resistance in wild birds and the possible interspecies transmission between humans, wildlife, livestock and the environment [9,32].

The role that non-food-borne exposure plays in the epidemiology of *C.jejuni* is currently not well defined. The aim of the current study was to perform a comprehensive population genetics and to explore the antimicrobial resistance of *C.jejuni* detected in Italian wild birds. In addition, we researched the presence of genomic features related to antimicrobial resistance between the wild bird isolates and estimate their importance from a public health perspective.

## 2. Results

We collected one hundred thirty-five *C.jejuni* from wild birds belonging to 15 species over a five period (2015 to 2019) in two geographically separate area in Italy. Wild birds included 97 pigeons (Columba livia), 7 magpie (Pica pica), 12 crows (Corvus sp), 2 greenfinch (Chloris chloris), 1 whitewagtail (Motacilla alba), 3 Eurasian Scops owl (Otus Scops), 2 starling (Sturnus vulgaris), 2 mallards (Anas platyrhynchos), 2 blackbirds (Turdus merula), 2 buzzard (Buteo buteo), 1 jackdaw (Corvus monedula), 1 jay (Cyanocitta), 1 seagull (Larus argentatus), 1 swift (Apus apus) and 1 trush (Psophocichla litsitsirupa) Table 1. 

One strain from a grey heron (Ardea cinerea) was not assigned to a specific *Campylobacter* spp. by specific PCR. The sequence of the16s rDNA gene resulted in potential identification of *Campylobacter volucris*. The genome assembly from the heron isolate confirmed the *C. volucris* species by Genome-to-Genome Distance Calculator (GGDC), an *in-silico* DNA-DNA hybridization method (DDH), using the DDH model “formula 2” as recommended for draft genomes. 

### 2.1. MLST and cgMLST

**Figure 1:**
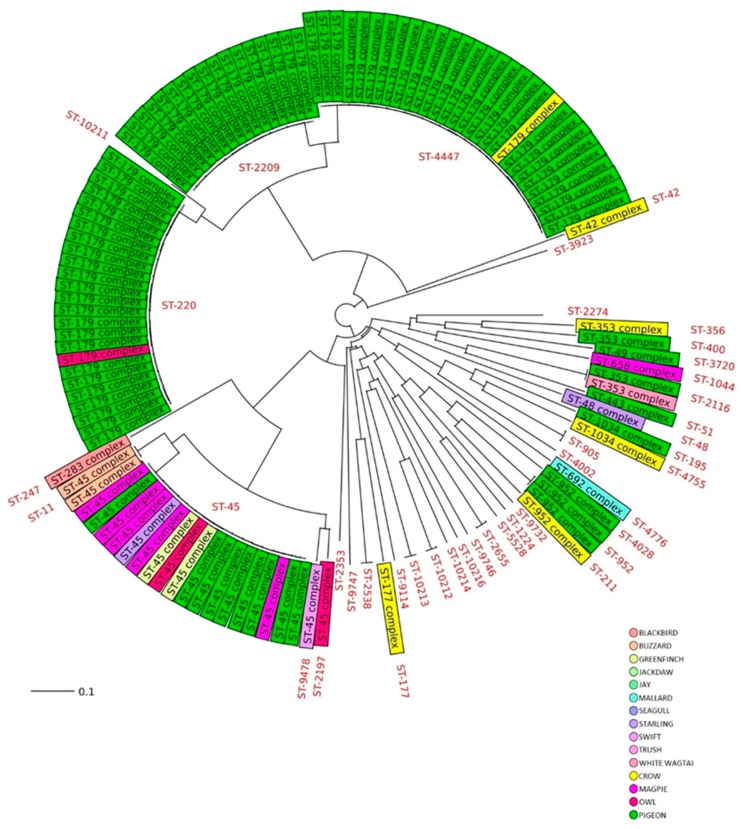
Neighbor joining tree of MLST profiles of *C.jejuni* isolated from Italian wild birds. In black the CCS are reported while the corresponding STS are indicated in red.

Among 135 *C.jejuni* characterized by MLST, 41 different STs and 13 CCs were identified (Table 1, Figure 1). Five novel STs, not previously represented in the PubMLST database (https://pubMLST.org/campylobacter), were identified (ST-10211, ST-10212, ST-10213, ST-10214, ST-10215 and ST-10216) (Figure 1). Eighteen STs resulted with not assigned clonal complex (Figure 1). ST-179 complex and ST-45 complex were the most frequently CCs found in the 53.3% and in the 16.3% of the isolates, respectively, confirming their association to the wild bird’s reservoir (Figure 1). They showed a wide geographical distribution and were found present throughout the study period. ST-45 was isolated from 4 different wild bird species (pigeons, magpies, starling and greenfinch) (Table 1). However, a very few numbers of STs were shared among different species. In particular, ST-2655 and ST-4447 were isolated in pigeons and crow; ST-2538 was isolated in one jay and in one thrush; ST-2116 in one pigeon and in one white wagtail, while ST-220 was isolated in many pigeons and in one owl (Table 1). Differently, a great number of STs revealed to be species-specific (Table 1). For example 9 STs were found only in crows; 14 different STs only in pigeons and 2 STs showed a host specificity toward mallards (Table 1, Figure 1). 

**Figure 2:**
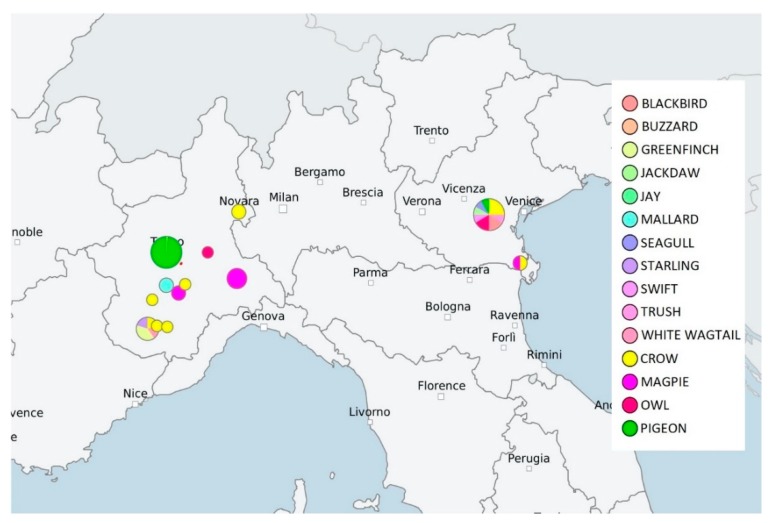
Geographic distribution of Italian wild birds analyzed. Geographical mapping of wild birds was obtained with Ridom SeqSphere+ v4.1.1 software using the geographical coordinates found from “city” entries. Colors within the mapped circle correspond to the wild bird species reported in the legend.

The wild bird’s geographic distribution in the North Italian regions is shown in Figure 2.

**Figure 3:**
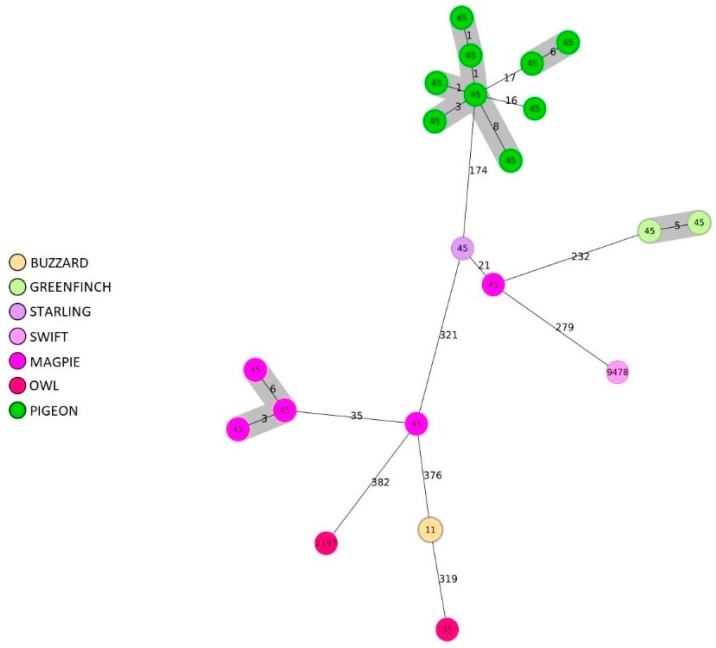
Minimum spanning tree (MST) generated for 22 isolates with ST-45 complex using the cgMLST approach. MST was calculated by pairwise comparison of 949 target genes with missing values ignored. Nodes correspond to unique profile and are colored according to wild bird species. The connecting lines between STs depict the number of allelic differences between them. Genotypes complexes with a distance of up to thirteen alleles are highlighted in grey.

**Figure 4:**
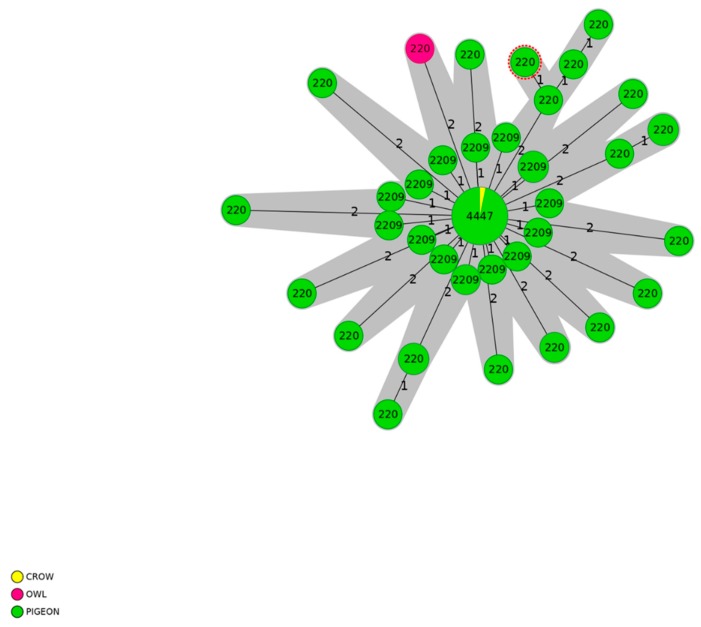
Minimum spanning tree (MST) generated for 72 isolates with ST-179 complex using the cgMLST approach. MST was calculated by pairwise comparison of 949 target genes with missing values ignored. Nodes correspond to unique profile and are colored according to wild bird species. The connecting lines between STs depict the number of allelic differences between them. Genotypes complexes with a distance of up to thirteen alleles are colored in grey.

The comparison of cgMLST allelic profiles using a cluster distance threshold of 13 different alleles from ST-45 complex and ST-179 complex revealed the presence of five and 1 clusters respectively (Figure 3 and Figure 4). In detail, for the ST-45 complex we observed two distinct clusters with 6 and 3 pigeons; 1 cluster with 2 greenfinches, 1 cluster with 3 magpie and 1 cluster composed of two buzzards (Figure 3). All the wild birds featured in the same clusters were captured in the same area.

Differently, the clonal ST-179 complex showed the presence of 70 pigeons, 1 crow and 1 owl (Figure 4). The pigeons were representative of all the districts analyzed in the study.

### 2.2. Antimicrobial Resistance Phenotypes

Altogether, 20% of the *C. jejuni* strains from wild birds were resistant to at least one of the six antibiotics tested. The MIC test revealed that 12.5% of the isolates were resistant to tetracycline, 10% of the strains showed resistance to nalidixic acid and ciprofloxacin, while 6.7% and 4.2% were resistant to streptomycin and erythromycin, respectively (Table 2). Few strains resulted resistant to gentamicin (2.5%) (Table 2). The 33.3% of the strains resistant to nalidixic acid, and the 25% of strains resistant to ciprofloxacin were assigned ST-179 complex. The 26.7% of *C. jejuni* resistant to tetracycline and the 16.7% of those resistant to ciprofloxacin and nalidixic acid were assigned to ST-353 complex. Finally, the 20% of *C. jejuni* resistant to tetracycline was assigned to ST-952 complex and the 13.3% to ST-45 complex. We identified seven *C. jejuni* multidrug resistance pattern (MDR), indicated in Figure 1. One *C. jejuni* strain isolated from a pigeon, showed resistance to all tested antibiotics (Figure 1). The most common MDR pattern (cipNatet) was observed for *C. jejuni* isolated from 2 pigeons, 1 crow and 1 white wagtail, while the other 2 (ciperygen) and (Naerystm) were observed in 2 pigeons (Figure 1).

### 2.3. Detection of Resistance Genes, Mutations and Levels of Concordance Among the Two Type of Resistances

The following genes of antimicrobial resistance were investigated: *tet(O), cmeA, cmeB, cmeC, cmeR, aad, oxa184, oxa61 and erm(B)*. The *tet (O)* gene, encoding tetracycline resistance ribosomal protection protein (TetO), was detected in 53.4% of wild bird strains. The multidrug efflux pump (CmeABC) and its regulator (CmeR), conferring resistance to a wide range of antimicrobials, were found in all the isolates; while *aad* gene, known to confer streptomycin resistance, was present in the 28.6% of wild bird isolates. The two major B-lactamase genes were differently observed, with a prevalence of *oxa-184* (86.7%) versus *oxa-61* (10%). Differently, *erm(B*) gene and point mutations on 23s rRNA regarded to be associated with erythromycin resistance, were not identified in any isolate.

*C. jejuni* strains showing resistance to quinolones and fluoroquinolones, were tested for point mutations in *gyrA* gene. Two types of mutations linked to ciprofloxacin and nalidixic acid resistance were detected in the analyzed wild bird populations on *gyrA* gene. 

In the detail, 25% possessed the C257T point mutation (2 pigeons and 1 crow), resulting in a T86I substitution in the *gyrA* gene, while 8.3% presented the A256G point mutation (1 pigeon) producing a T86V substitution. Weak correlations were found for phenotypic and genotypic resistances, reaching levels of concordance of 23.3%, 23.4% and of 30% for streptomycin, tetracycline and quinolones/fluoroquinolones, respectively (Table 2). 

## 3. Discussion

Reducing the occurrence of campylobacteriosis is a food safety issue of high priority in European Union; however, continuing invasion on wildlife habitat by humans through a massive urbanization and intensive agriculture increases the opportunities for contact between wild birds, domestic animals, and people [9]. Wild birds, being natural reservoir of *Campylobacter*, thus represent a serious risk for public health, either directly through the consumption of wild bird game meat, or indirectly, by disseminating *Campylobacter* into the environment and domestic livestock [14]. The aim of this study was to assess the genomic characterization of circulating wild bird species in Italy and indagate their capability to disseminate in the environment important antimicrobials factors, in order to evaluate their influence on public health. Wild birds are usually considered to have a marginal role in human illness. However, specific STs and generalist lineages of *C. jejuni* found in hospitalized patients with gastroenteritis have been reported in several species of wild bird [13,15,16,17,21], indicating their potentiality as reservoirs of human infection. ST-45 and ST-267, isolated in humans, have been found among both blackbird and chicken isolates from Sweden, magpies, greenfinches, starling, pigeons, owl and blackbird in Italy [26,33,34]. In a previous study [26] other STs (ST-48, 2116 and 1044) from Italian human patients, were shared with starling, magpies and white wagtail circulating in Italy. Differently, the high occurrence (73.2%) of species-specific STs, would confirm the existence of particular *C. jejuni* lineages for specific wild bird species. ST-1224 that we isolated in one magpie, has only been found in wild birds from USA and in environmental water in Canada, confirming on one side a species-specificity, on the other side the bacteria dissemination in environmental waters through wild birds acting as vectors [35,36]. Moreover, ST-2353 isolated in a seagull, has been found only in a gull in New Zealand in 2008 so far, as well as in environmental water in the same area (www.pubMLST.org), reinforcing the idea that gulls are not a major source of *C. jejuni* in human infections. In our study, two major CCs were reported in 53.3% (ST-179 complex) and in 16.3% (ST-45 complex) of wild bird population; thus, in order to analyze the genomic diversity within the most detected clonal complexes, a comparative genomics using cgMLST was performed. The minimum spanning tree based on the cgMLST of ST-179 complex clearly indicates that although the isolates clustered in three groups defined by different STs, they form a cohesive single population with only 1 or 2 allelic differences. Furthermore, in addition to 70 pigeons, we found a crow and an owl within the cluster, confirming the idea that these STs are commonly shared between different animal host species. In detail, the crow belonged to the same town of the pigeons, while the owl was collected from a sampling site 400 km away. Otherwise, the ST-45 complex, comprised seven different species, nonetheless the isolates were more closely related to those of the same species, independently from the sampling site or time of isolation. In our study, the sampling size and the variety of species were defined by the use of passive surveillance without active case finding, therefore, it is not possible to make a general conclusion on the whole wild bird population. Nonetheless, the results aided several other studies and seem to corroborate the idea that wild birds not only can disseminate *C. jejuni* in different regions, but they can harbor different lineages of *C. jejuni.* Our results are in line with a recent study demonstrating the existence of certain sub lineages of ST-45 forming genetically isolated clades containing *C. jejuni* strains with extremely similar genomes regardless of time and location of sampling [27]. In addition to the highly mobile nature of many wild bird species to disseminate bacteria in the environment [32], the World Health Organization in 2017 (World Health Organization, 2017) showed that many of these microorganisms exhibited resistance to antimicrobials considered of highest importance to human medicine [37,38]. Interestingly, *C.jejuni* strains isolated in this study showed a moderate percentage resistance to tetracycline, to ciprofloxacin and nalidixic acid, suggesting that the antimicrobial resistance could be acquired by horizontal gene transfer [39] or, to the usage antibiotics as a therapeutic agent in dairy cattle and poultry farms [40]. Lower resistance rates have been observed for streptomycin, erythromycin and gentamicin. The prevalence observed in our study is partially in line with data obtained by Aksomaitiene at al. [41] for tetracycline and erythromycin in Lithuania. A low number of streptomycin-resistant *C.jejuni* from wild birds has been previously described [42]. In contrast, a worrying resistance rate for quinolones, fluoroquinolones and tetracyclines has been detected in the same *C. jejuni* strains. Krawiek et al, shows higher level of resistance, respect to our data, for all antibiotics with the exception of nalidixic acid, slightly lower than that reported from us probably related to different environmental features [24]. 

We also studied our wild birds in the context of MDR, defined as resistance to at least three different antimicrobials [43]. Antimicrobial resistance is a complex problem and the wildlife role could be underestimated. In our dataset we found only 5.2% of wild birds resistant to three different antibiotic classes. However, one *C.jejuni* from a bird usually living strictly with human people (pigeon) showed a worrying MDR to all the antimicrobials tested in the study, reinforcing the idea that wild birds represent a real risk in spreading resistant bacteria. Wild bird studies depend on opportunistic sample collection; therefore, some birds are more likely to be sampled than others, as happen in this study with the pigeons that are the majority in this set of wild birds analyzed.

*C.jejuni* isolates resistant to cip and Na were screened for the presence of the mutations in the quinolone resistance-determining regions (QRDR) of the *gyrA* gene. We found T86I amino acid substitution as the most common (25% of pigeons and crow isolates), followed by only a pigeon with T86V aminoacid substitution. The rest of wild birds resulted without mutations, confirming other mechanisms of resistance such as pump efflux system, as reported previously [44]. We found the genes involved in this multidrug CmeABC efflux system, together to his repressor cmeR, present in all wild bird isolates. However, our wild strains miss the known genetic mechanisms for the ery resistance, implying that resistance to ery could be the result of another unknown system in our strains. 

These findings are in line with the study of Yao et al. that revealed the existence of a “super” CmeABC variant used by these bacteria to enhance multidrug resistance, desensitizing *Campylobacter* to many antimicrobials and conferring a high-level resistance to fluoroquinolone in association with the C257T mutation in *gyrA* [45]. 

We screened for other anti-resistant factors such as *blaoxa-184* and *blaoxa-61* gene, described to confer beta-lactam resistance in *C.jejuni* strains by Alfredson et al 2005 [46]. We found the 86.7% and the 10% of the genes respectively presents, in line with other studies [45,47]. This is worth to investigate further because this antimicrobial class usually is not used for the *Campylobacter* infections. Finally, in this study we found a low correlation between phenotypic resistance to tetracycline, streptomycin and quinolones/fluoroquinolones and the existence of resistance genes or nucleotide polymorphisms. 

In conclusion, our study demonstrated that wild birds are mostly colonized by host-adapted STs, possessing a minor set of STs shared with further hosts, including poultry, livestock and humans. Although, we found a low and moderate prevalence of antibiotic resistance in our data set, it is extremely important to monitor the situation also in wild birds. Understanding of the prevalence of these potentially dangerous bacteria in wild birds is necessary to guide public health policies about the control of foodborne illness in humans. Similarly, the occurrence of resistant bacteria in wildlife has been detected in many animal species across different geographical areas, highlighting the importance and complexity of wild animals, in the transmission of resistant bacteria, even if they are not usually exposed directly to antimicrobials. Thus, continued surveillance of multi-resistant bacteria in wild animals is warranted.

## 4. Materials and Methods 

### 4.1. Bacterial Strains and Species Identification

A total of 135 wild bird isolates from Piedmont and Veneto regions were included in the study. In particular, 67/135 were previously analyzed for antimicrobial susceptibility [44] and added to the others sixtyeight. Strains were isolated during five years (2015–2019) via passive surveillance monitoring by the Istituti Zooprofilattici Sperimenatali (IIZZSS) network and/or sent to the National Laboratory Reference of *Campylobacter* (NRL, http://www.izs.it/IZS/Eccellenza/Centri_nazionali/LNR-Campylobacter), for species identification or molecular characterization. The strains were cultured on Columbia blood agar plates in microaerobic atmosphere at 42 °C for 48h and stored at −80 °C in Microbank ™ until further analysis. After an initial phenotypic characterization, suspected colonies confirmed as thermotolerant *Campylobacter* and identified to species level using a multiplex and a simplex PCR, as described previously (Marotta 2019). Strains used as positive controls were *Campylobacter coli* NCTC 11353, *Campylobacter fetus* ATCC 19438, *Campylobacter jejuni* ATCC 33291, *Campylobacter upsaliensis* NCTC 11541 and *Campylobacter lari* NCTC 11552. DNA was extracted using Maxwell instrument (Promega Corporation, Madison, WI, USA) according to the manufacturer’s instructions and quantified using a Nanodrop Spectrophotometer (Nanodrop Technologies, Celbio Srl., Milan, Italy).

### 4.2. Sequence Analysis and Identification of Antibiotic Resistance Genes

Total genomic DNA was used to prepare sequencing libraries using Nextera XT Library Preparation Kit (Illumina, Inc., San Diego, CA, USA). The libraries were then sequenced using Illumina NextSeq 500 sequencer. Sequence reads (150-bp, pair-end) were demultiplexed and the adapters were removed. Subsequently the reads were trimmed with Trimmomatic tool (version 0.36) and de novo assembled using SPAdes version 3.11.1 with the ‘careful’ option selected [48]. The sequence reads generated in this study were deposited in NCBI Sequence Read Archive (SRA) in Bioproject PRJNA623711. Additionally, 15 previously published sequences used in the analysis can be found in NCBI Bioproject PRJNA510785 [49].

*C.jejuni* genome assemblies, were genotyped by using MLST and cgMLST. The assemblies were also investigated for the genomic AMR traits. Gene-by-gene analysis was performed using the SeqSphere+ v4.1.1 software (RidomGmbH, Münster, Germany). The MLST profiles were assigned using a *C.jejuni/coli* task template MLST 7 loci, schema available at https://pubmlst.org/campylobacter/ accessible through in Ridom SeqSphere+ software. The cgMLST profiles were assigned using *C.jejuni* task template with 949 target core genomes available in the Ridom SeqSphere+ database (https://www.ridom.de/seqsphere/u/Task_Template_Sphere.html). A Neighbor joining tree was generated for MLST profiles, while a Minimum spanning tree (MST) was generated by pairwise comparison of cgMLST target genes using default settings parameters of Ridom. Missing alleles were ignored in the pairwise comparisons. 

AMR genes were identified in silico using PointFinder v. 3.1.0 and ABRicate v. 0.8 (https://github.com/tseemann/abricate/) by querying the publicly available Comprehensive Antibiotic Resistance Database (CARD) [50,51]. Prokka v1.13 [52] was used to annotate the assemblies and gyrA sequences were extracted applying the query_pan_genome function in Roary v3.12.0 [53]. gyrA genes were aligned using Uniprot UGENE v1.18.0 [54], from which the gene variants were identified. Only mutations in the quinolone resistance-determining region (QRDR) of gyrA were assessed to be the determinants of resistance, as only these loci have been linked with phenotypic resistance to quinolones. In particular we analyzed the amino acid changes at position 86.

### 4.3. Antimicrobial Susceptibility

A total of 68/135 wild bird strains were subjected to the antimicrobial test and added to the previously analyzed [44]. Susceptibility to antimicrobials was evaluated with the microdilution method using the Sensititre automated system (TREK Diagnostic Systems, Venice, Italy). Colonies were sub cultured on Columbia agar for 24 hours and then seeded in Mueller Hinton Broth supplemented with blood (Oxoid, Basingstoke, UK). Subsequently, they were dispensed into Eucamp2 microtiter plates (TREK Diagnostic Systems, Venice, Italy), containing known scalar concentrations of the following antibiotics, indicating the distribution % of Minimum Inhibitory Concentration (MIC): ciprofloxacin (0.12–16 μg/mL), erythromycin (1–128 μg/mL), gentamicin (0.12–16 μg/mL), nalidixic acid (1–64 μg/mL), streptomycin (0.25–16 μg/mL), and tetracycline (0.5–64 μg/mL). After inoculation, the plates were incubated at 42 °C in microaerobic atmosphere for 24 h and then screened. *Campylobacter jejuni* strain NCTC 11351 was used as control. The strains were classified as resistant (R), and susceptible (S) to the examined antimicrobials based on MIC breakpoints, by using Swin v3.3 Software (Thermo Fisher Scientific) in accordance with the epidemiological cutoff values (ECOFFs) as defined by EUCAST (European Committee on antimicrobial breakpoints) (www.eucast.org) to interpret their antimicrobial susceptibilities. MIC breakpoints of resistance were ≥0.5 μg/mL for ciprofloxacin, ≥4 μg/mL for streptomycin, ≥4 μg/mL for erythromycin, ≥2 μg/mL for gentamicin, ≥16 μg/mL for nalidixic acid and ≥1 μg/mL for tetracycline. Details of the wild bird isolates are summarized in supplementary Table S1.

## Figures and Tables

**Table 1:** *C.jejuni* wild bird isolates with MLST, resistances phenotype, resistances genotype and MDR profiles.

				Resistance Phenotypes (No. Isolates)						Resistance Genotypes						MDR			
Family	Species	ST (No. Isolates)	CC	gen	stm	cip	Na	ery	tet	*aad*	*blaOXA-61*	*blaOXA-184*	*gyrA*	*Tet(O)*	*cmeA,B,C,R*	cipNatet	cipNateterystmCipEGG	ciperygen	Naerystm
Accipitridae	Common Buzzard *(Buteo buteo)*	11 (2)	45																
Anatidae	Mallard *(Anas platyrhynchos)*	4776 (1)	ST-692 complex																
		4002 (1)	na																
Columbidae	Pigeon *(Columba livia)*	10211 * (1)	na																
		10212 * (2)	na						1										
		10214 * (1)	na																
		1956 (1)	ST-1034 complex																
		2116 (1)	ST-353 complex																
		220 (24)	ST-179 complex				1												
		2209 (17)	ST-179 complex				1												
		2274 (1)	na																
		2665 (1)	na																
		3720 (1)	ST-49 complex																
		3923 (1)	na																
		400 (1)	ST-353 complex																
		4028 (1)	ST-952 complex																
		4447 (29)	ST-179 complex	2	2	3	3	3	1										
		45 (9)	ST-45 complex		1				1										
		51 (1)	ST-443 complex																
		5528 (1)	na																
		905 (2)	na																
		952 (2)	ST-952 complex		2				2										
Corvidae	Crow *(Corvus sp.)*	10213 * (2)	na																
		10216 * (1)	na																
		177 (1)	ST-177 complex																
		2655 (1)	na																
		9732 (1)	na																
		4755 (1)	ST-1034 complex																
		356 (1)	ST-353 complex																
		2111 (1)	ST-952 complex																
		4447 (1)	ST-179 complex																
		9114 (1)	na																
		42 (1)	ST-42 complex																
	Magpie *(Pica pica)*	45 (5)	ST-45 complex		1		1												
		1224 (1)	Na																
		1044 (1)	ST-658 complex																
	Jackdaw *(Corvus onedula)*	9746 (1)	na																
	Eurasian jay *(Cyanocitta)*	2538 (1)	na																
Strigidae	Eurasian Scops Owl *(Otus scops)*	2197 (1)	ST-45 complex																
		220 (1)	ST-179 complex																
		45 (1)	ST-45 complex																
Fringillidae	Greenfinch *(Chloris chloris)*	45 (2)	ST-45 complex			2	1		1										
Motacillidae	Whitewagtail *(Motacilla alba)*	2116 (1)	ST-353 complex																
Sturnidae	European starling *(Sturnus vulgaris)*	45 (1)	ST-45 complex																
		48 (1)	ST-48 complex																
Turdidae	Blackbirds *(Turdus merula)*	9747 (1)	na																
		267 (1)	ST-283 complex																
	Thrush *(Psophocichla litsitsirupa)*	2538 (1)	na																
Laridae	Seagul *(Larus argentatus)*	2353 (1)	na																
Apodidae	Swift *(Apus apus)*	9478 (1)	ST-45 complex																

gen = gentamicin; stm = streptomycin; cip = ciprofloxacin; Na = nalidixic acid; ery = erytromicin; tet = tetracycline.* = novel ST in this study; na = not assigned to a clonal complex.

**Table 2:** Comparison of genotypic and phenotypic resistance to antibiotics in *C. jejuni* isolated from Italian wild birds.

Antibiotic Class	Antibiotics	Genes ^a^	% Resistances (Phenotypes) ^b^	% Resistance (Genotypes) ^c^	Concordance rate ^d^
Aminoglycosides	Gentamicin (gen)	-	2.5		
	Streptomycin (stm)	*aad*	6.7	28.6	23.3
Beta-lactams ^e^	-	*blaOXA-61, blaOXA-184*	-	10, 86.7	-
Fluoroquinolones/ Quinolones	Ciprofloxacin (cip)/ Nalidixic acid (Na)	*gyrA*	10	33.3	30.0
Macrolides	Erytromicin (ery)	*erm(B), 23S rRNA*	4.2	-	-
Tetracyclines	Tertracycline (tet)	*Tet(O)*	12.5	53.3	23.4
Multidrug CmeABC efflux system and cmeR		*cmeA, cmeB, cmeC, cmeR*	-	100, 100, 99.2, 100	-

^a^ Accession numbers from the resistant genes can be accessed through the database Card https://card.mcmaster.ca/, point mutations were searched according to point finder database (https://bitbucket.org/genomicepidemiology/pointfinder_db/src/master/campylobacter/resistens-overview.txt); ^b^ Percentages of isolates expressing the resistance phenotype for the corresponding antibiotic; ^c^ Percentages of isolates expressing the resistance phenotype for the corresponding antibiotic, that have the indicated gene; ^d^ Concordance rate among the two resistances (%), ^e^ Antibiotic class not tested for resistance phenotype.

## Supplementary Materials

Supplementary materials are available online at https://www.mdpi.com/2076-0817/9/4/304/s1. Table S1: Minimum Inhibitory Concentration (MIC) for the isolates tested.
