# Supplementary material for: Molecular Characterization and Antimicrobial Susceptibility of C. jejuni Isolates from Italian Wild Bird Populations
Source: Pathogens. 2020 Apr 20;9(4):304. doi: 10.3390/pathogens9040304 (PMC7238051; doi:10.3390/pathogens9040304)
Supplement: Supplementary file 1 [file pathogens-09-00304-s001.pdf]

Table S1. Minimum Inhibitory Concentration (MIC) for the isolates tested.

Isolate	Wild bird	Ciprofloxacin (0.12-16 µg/ml)*	Erythromycin (1-128 µg/ml)*	Gentamicin (0.12- 16 µg/ml)*	Nalidixic Acid (1-64 µg/ml)*	Streptomycin (0.25- 16 µg/ml)*	Tetracycline (0.5-64 µg/ml)*
2015-TE-14491-1-1	WHITE WAGTAIL	0.12	64	1	4	2	64
2015-TE-14493-1-1	STARLING	0.12	1	1	4	2	0.5
2015-TE-17432-1-1	MAGPIE	0.12	0.5	0.5	8	1	0.5
2015-TE-17433-1-1	MAGPIE	0.12	0.5	0.5	8	1	0.5
2015-TE-17434-1-1	MAGPIE	0.25	0.5	2	32	8	1
2015-TE-17435-1-1	MAGPIE	0.25	1	2	8	4	1
2015-TE-17436-1-1	MAGPIE	0.12	1	1	8	2	1
2015-TE-18869-1-1	CROW	0.06	0.5	0.5	4	2	0.25
2015-TE-18871-1-1	CROW	0.06	0.5	0.5	4	2	0.25
2015-TE-20126-1-1	OWL	0.12	1	0.5	8	2	0.5
2015-TE-23983-1-1	GREENFINCH	4	1	1	16	2	0.5
2015-TE-23984-1-1	GREENFINCH	8	1	0.5	8	2	64
2015-TE-28365-1-1	PIGEON	16	128	16	2	0.5	2
2015-TE-30001-1-1	PIGEON	16	128	16	64	16	64
2015-TE-31718-1-1	PIGEON	0.12	1	0.5	4	16	16
2015-TE-31719-1-1	PIGEON	0.12	1	0.5	4	16	16
2015-TE-33740-1-1	PIGEON	0.12	1	1	8	16	8
2016-TE-10309-1-1	PIGEON	0.12	1	0.25	1	1	0.5
2016-TE-10310-1-1	PIGEON	0.12	1	0.25	4	1	0.5
2016-TE-12094-1-1	PIGEON	16	1	0.5	64	1	0.5
2016-TE-12096-1-1	PIGEON	0.12	1	0.5	4	2	0.5
2016-TE-12097-1-1	PIGEON	0.12	1	0.5	2	2	0.5
2016-TE-12816-1-1	PIGEON	0.25	1	0.5	4	1	0.5
2016-TE-18387-1-1	PIGEON	0.25	1	0.5	4	1	0.5
2016-TE-20375-1-1	CROW	0.12	1	0.12	1	0.25	0.5
2016-TE-22529-1-1	PIGEON	0.12	1	0.5	4	2	0.5
2016-TE-24037-1-1	PIGEON	8	128	0.5	64	2	0.5
2016-TE-24038-1-1	PIGEON	16	1	0.5	64	1	32

2016-TE-24471-1-1	CROW	0.12	1	1	8	2	0.5
2016-TE-25584-1-1	CROW	0.12	1	0.5	8	2	0.5
2016-TE-25585-1-1	PIGEON	0.12	1	0.12	4	2	0.5
2016-TE-25586-1-1	PIGEON	0.12	1	0.5	8	2	0.5
2016-TE-27119-1-1	PIGEON	0.12	1	1	4	4	0.5
2016-TE-27121-1-1	PIGEON	0.12	1	1	4	2	0.5
2016-TE-27123-1-1	PIGEON	0.12	1	0.5	4	2	0.5
2016-TE-27124-1-1	PIGEON	0.12	1	0.5	4	2	0.5
2016-TE-27127-1-1	PIGEON	0.12	1	0.5	4	2	0.5
2016-TE-6009-1-1	PIGEON	0.12	1	1	4	4	64
2016-TE-6010-1-1	PIGEON	0.25	1	1	8	4	0.5
2016-TE-6832-1-1	PIGEON	0.25	1	1	8	2	0.5
2016-TE-6834-1-1	PIGEON	0.25	1	1	8	4	0.5
2016-TE-8199-1-1	PIGEON	0.12	1	0.25	16	1	0.5
2016-TE-8597-1-1	PIGEON	0.25	1	2	8	8	0.5
2017.TE.27617.1.1	PIGEON	16	1	0.5	64	2	64
2017.TE.27619.1.1	PIGEON	0.12	1	0.5	4	1	0.5
2017.TE-27620.1.1	PIGEON	0.12	1	0.25	4	1	0.5
2017-TE-12276-1-1	PIGEON	0.12	1	0.5	8	2	16
2017-TE-12278-1-1	MAGPIE	0.12	1	0.5	8	2	0.5
2017-TE-12279-1-1	PIGEON	0.12	1	0.12	1	0.25	0.5
2017-TE-16478-1-1	PIGEON	0.12	1	0.12	4	1	0.5
2017-TE-16479-1-1	PIGEON	0.12	1	0.25	4	1	0.5
2017-TE-16480-1-1	PIGEON	0.12	1	0.12	4	0.5	0.5
2017-TE-16481-1-1	PIGEON	0.12	1	0.5	4	2	0.5
2017-TE-17866-1-1	MALLARD	0.12	1	0.5	8	2	0.5
2017-TE-17868-1-1	MALLARD	0.12	1	0.5	8	2	0.5
2017-TE-19477-1-1	PIGEON	0.12	1	0.5	4	2	0.5
2017-TE-19479-1-1	PIGEON	0.12	1	0.25	4	1	0.5
2017-TE-19481-1-1	PIGEON	0.12	1	0.12	4	1	0.5
2017-TE-19486-1-1	PIGEON	0.12	1	0.5	8	2	0.5
2017-TE-19487-1-1	PIGEON	0.12	1	0.25	4	2	0.5

2017-TE-3069-1-1	PIGEON	0.12	1	0.5	8	4	0.5
2017-TE-4824-1-1	PIGEON	0.12	1	0.25	4	1	0.5
2017-TE-6838-1-1	PIGEON	0.12	1	0.5	4	1	64
2017-TE-6839-1-1	PIGEON	0.12	1	1	8	4	64
2017-TE-6840-1-1	PIGEON	0.12	1	0.5	8	2	0.5
2017-TE-6841-1-1	PIGEON	0.12	1	0.12	8	2	0.5
2018-TE-11848-1-1	PIGEON	0.12	1	0.12	2	0.25	0.5
2018-TE-11849-1-1	PIGEON	0.12	1	0.12	4	0.5	0.5
2018-TE-12930-1-2	PIGEON	0.12	1	0.12	4	0.5	0.5
2018-TE-16331-1-1	STARLING	0.12	1	1	8	2	0.5
2018-TE-21418-1-1	PIGEON	8	1	0.5	64	1	0.5
2018-TE-23305-1-1	PIGEON	0.25	1	0.12	16	4	0.5
2018-TE-23306-1-1	PIGEON	0.12	1	0.25	4	2	0.5
2018-TE-23307-1-1	PIGEON	0.12	1	1	4	2	0.5
2018-TE-23310-1-1	PIGEON	0.12	1	0.5	4	1	0.5
2018-TE-23311-1-1	PIGEON	0.12	1	0.5	4	2	0.5
2018-TE-23312-1-1	PIGEON	0.12	1	0.25	32	1	1
2018-TE-23313-1-1	PIGEON	0.12	1	1	4	2	0.5
2018-TE-23314-1-1	PIGEON	0.12	1	0.5	4	1	0.5
2018-TE-23316-1-1	PIGEON	0.12	1	0.5	4	1	0.5
2018-TE-24535-1-1	PIGEON	0.12	1	0.5	4	1	<=0.5
2018-TE-24538-1-1	PIGEON	0.12	1	0.5	4	2	<=0.5
2018-TE-5897-1-1	BUZZARD	0.12	1	0.25	4	2	0.5
2018-TE-5898-1-1	BUZZARD	0.12	1	0.25	4	2	1
2018-TE-6191-1-1	CROW	16	1	1	64	2	64
2018-TE-6192-1-1	PIGEON	0.12	1	0.25	2	1	0.5
2018-TE-6193-1-1	CROW	0.12	1	0.5	4	2	0.5
2018-TE-6194-1-1	PIGEON	0.12	1	0.5	8	2	0.5
2018-TE-6196-1-1	PIGEON	0.12	1	0.5	4	2	0.5
2018-TE-7336-1-1	PIGEON	0.12	1	0.5	4	2	0.5
2018-TE-7337-1-1	PIGEON	0.12	1	0.5	4	2	0.5
2018-TE-7338-1-1	PIGEON	0.12	1	0.5	4	2	0.5

2019.TE.25696.1.10	PIGEON	0.12	1	0.5	4	2	0.5
2019.TE.25696.1.11	PIGEON	0.25	1	0.5	8	4	0.5
2019.TE.25696.1.12	PIGEON	0.25	1	0.5	8	2	0.5
2019.TE.25696.1.15	PIGEON	0.12	1	0.5	4	1	0.5
2019.TE.25696.1.16	PIGEON	0.12	1	0.5	4	2	0.5
2019.TE.25696.1.17	PIGEON	0.12	1	0.12	2	0.5	0.5
2019.TE.25696.1.18	PIGEON	0.12	1	0.5	4	1	0.5
2019.TE.25696.1.19	PIGEON	0.12	1	0.5	4	1	0.5
2019.TE.25696.1.20	PIGEON	0.12	1	0.12	2	0.5	0.5
2019.TE.22020-1-1	PIGEON	4	1	0.5	64	1	32
2019.TE.25696.1.22	PIGEON	0.12	1	0.5	4	1	0.5
2019.TE.25696.1.23	PIGEON	0.12	1	0.25	8	1	0.5
2019.TE.25696.1.24	PIGEON	0.12	1	0.5	8	16	0.5
2019.TE.25696.1.25	PIGEON	0.12	1	0.25	4	1	0.5
2019.TE.25696.1.26	PIGEON	0.12	1	0.5	4	1	0.5
2019.TE.25696.1.5	PIGEON	0.12	128	4	64	8	0.5
2019.TE.25696.1.9	PIGEON	0.12	1	0.5	8	2	0.5
2019-TE-13423-1-1	PIGEON	0.12	1	0.25	4	1	0.5
2019-TE-3612-1-1	CROW	16	1	1	64	4	0.5
2019-TE-4614-1-1	PIGEON	0.12	1	1	4	4	0.5
2019-TE-4615-1-1	PIGEON	0.12	1	0.25	4	1	0.5
2019-TE-5079-1-1	PIGEON	0.12	1	0.5	8	4	0.5
2019-TE-5080-1-1	PIGEON	0.25	1	1	8	4	0.5
2019-TE-5081-1-1	PIGEON	0.25	1	1	8	4	0.5
2019-TE-5493-1-1	PIGEON	0.25	1	0.5	8	2	0.5
2019-TE-6597-1-1	PIGEON	0.12	1	0.25	4	1	0.5
2019-TE-6599-1-1	PIGEON	0.12	1	0.5	4	2	0.5
2019-TE-8512-1-1	PIGEON	0.25	1	0.5	8	2	0.5

*=Minimum Inhibitory Concentration (MIC)
